# Asp305Gly mutation improved the activity and stability of the styrene monooxygenase for efficient epoxide production in *Pseudomonas putida* KT2440

**DOI:** 10.1186/s12934-019-1065-5

**Published:** 2019-01-24

**Authors:** Chunlin Tan, Xian Zhang, Zhijing Zhu, Meijuan Xu, Taowei Yang, Tolbert Osire, Shangtian Yang, Zhiming Rao

**Affiliations:** 1 The Key Laboratory of Industrial Biotechnology, Ministry of Education, School of Biotechnology, Jiangnan University, 1800 Lihu Road, Wuxi, 214122 Jiangsu China; 2 The School of Digital Media, Jiangnan University, Wuxi, 214122 China; 3 Department of Chemical and Biomolecular Engineering, The Ohio State University, Columbus, OH 43210 USA

**Keywords:** Styrene monooxygenase, Biocatalysis, Site-saturation mutagenesis, Epoxide

## Abstract

**Background:**

Styrene monooxygenase (SMO) catalyzes the first step of aromatic alkene degradation yielding the corresponding epoxides. Because of its broad spectrum of substrates, the enzyme harbors a great potential for an application in medicine and chemical industries.

**Results:**

In this study, we achieved higher enzymatic activity and better stability towards styrene by enlarging the ligand entrance tunnel and improving the hydrophobicity through error-prone PCR and site-saturation mutagenesis. It was found that Asp305 (D305) hindered the entrance of the FAD cofactor according to the model analysis. Therefore, substitution with amino acids possessing shorter side chains, like glycine, opened the entrance tunnel and resulted in up to 2.7 times higher activity compared to the wild-type enzyme. The half-lives of thermal inactivation for the variant D305G at 60 °C was 28.9 h compared to only 3.2 h of the wild type SMO. Moreover, overexpression of SMO in *Pseudomonas putida* KT2440 with NADH regeneration was carried out in order to improve biotransformation efficiency for epoxide production. A hexadecane/buffer (v/v) biphasic system was applied in order to minimize the inactivation effect of high substrate concentrations on the SMO enzyme. Finally, SMO activities of 190 U/g CDW were measured and a total amount of 20.5 mM (*S*)-styrene oxide were obtained after 8 h.

**Conclusions:**

This study offers an alternative strategy for improved SMO expression and provides an efficient biocatalytic system for epoxide production via engineering the entrance tunnel of the enzyme’s active site.

**Electronic supplementary material:**

The online version of this article (10.1186/s12934-019-1065-5) contains supplementary material, which is available to authorized users.

## Background

Chiral epoxides are a class of high value-added synthons or intermediates with a broad scope of market demand and application in the production of pharmaceuticals, agrochemicals, as well as versatile fine chemicals. Conventionally, epoxides are produced by chemical catalysis. However, this approach presents many shortcomings such as harsh reaction conditions, low selectivity, and many by-products, thus causing significant environmental pollution. Considerable efforts and several enzymatic approaches have been designed by synthetic chemists to overcome these challenges and thus find environmentally friendly alternatives [1–3].

Styrene monooxygenase (SMO, EC: 1.14.14.11), a flavin-dependent enzyme complex consisting of two components (an oxygenase subunit of StyA and a reductase subunit of StyB) [4], was found in several *Pseudomonas* species and has been used successfully to catalyze styrene to (*S*)-styrene oxide, a valuable chiral intermediate for synthesizing some important pharmaceuticals, such as cilastatin and levamisole [5, 6]. The SMO, which is naturally involved in the upper catabolic pathway of styrene degradation, shows excellent enantioselectivity in the epoxidation of styrene to (*S*)-styrene epoxide with > 99% ee [7]. This has attracted many efforts to the synthesis of chiral epoxides [8–10]. For example, SMOs from *Pseudomonas fluorescens* ST and *Pseudomonas* sp. VLB120 have been used to convert conjugated styrene derivatives as well as aromatic sulfides [11, 12]. Therefore, recombinant SMO expression in *E. coli* for use in biocatalysis has been investigated by several studies [13].

However, low substrate solubility, low enzyme activity and the consumption of redox equivalents by the SMO are the primary factors restricting its application for epoxide biosynthesis. Several approaches have been developed to solve this problem during the past few decades [14–16]. Wu et al. produced (*S*)-vicinal diols with more than 99% ee in recombinant *E. coli* using 10% ethanol as cosolvent [7]. Furthermore, Hu et al. used a biphasic *n*-hexanol buffer system to improve the epoxide hydrolase-catalyzed kinetic resolution [15]. The use of a biphasic system in tissue-mediated biotransformation was mainly aimed at overcoming the low water solubilities of substrates and inhibitory effects of products [17, 18].

Moreover, the strategies for screening high enzyme activity variants and exploring the efficient biotransformation are also essential. Therefore, Gursky et al. previously undertook the random-library screening approach based on color (indigo) formation [4]. However, this method cannot be easily adapted to detect changes in the substrate preferences of SMOs [19]. Another study on the engineering of the SMO from *P. putida* CA-3 has been performed by screening an error-prone PCR (epPCR) library using the indigo assay [4]. However, the method carries the risk of generating mutants with increased activity only towards the analog substrate indigo, but not the target substrate styrene [20]. Assays based on the reaction of 1,2-diols or on the reaction of epoxides with 4-nitrobenzyl-pyridine might be suitable for this purpose [19, 21]. The NBP assay provides a sensitive method for detection on paper and thin-layer chromatograms and for quantitative colorimetric analysis of many epoxides of biological, chemical and environmental significance [22]. In this study, a new high throughput screening method based on NBP assay and error prone PCR was established for improved screening of high enzyme activity mutants. Such mutants harbor a great potential for an application in epoxide biosynthesis.

**Fig. 1:**
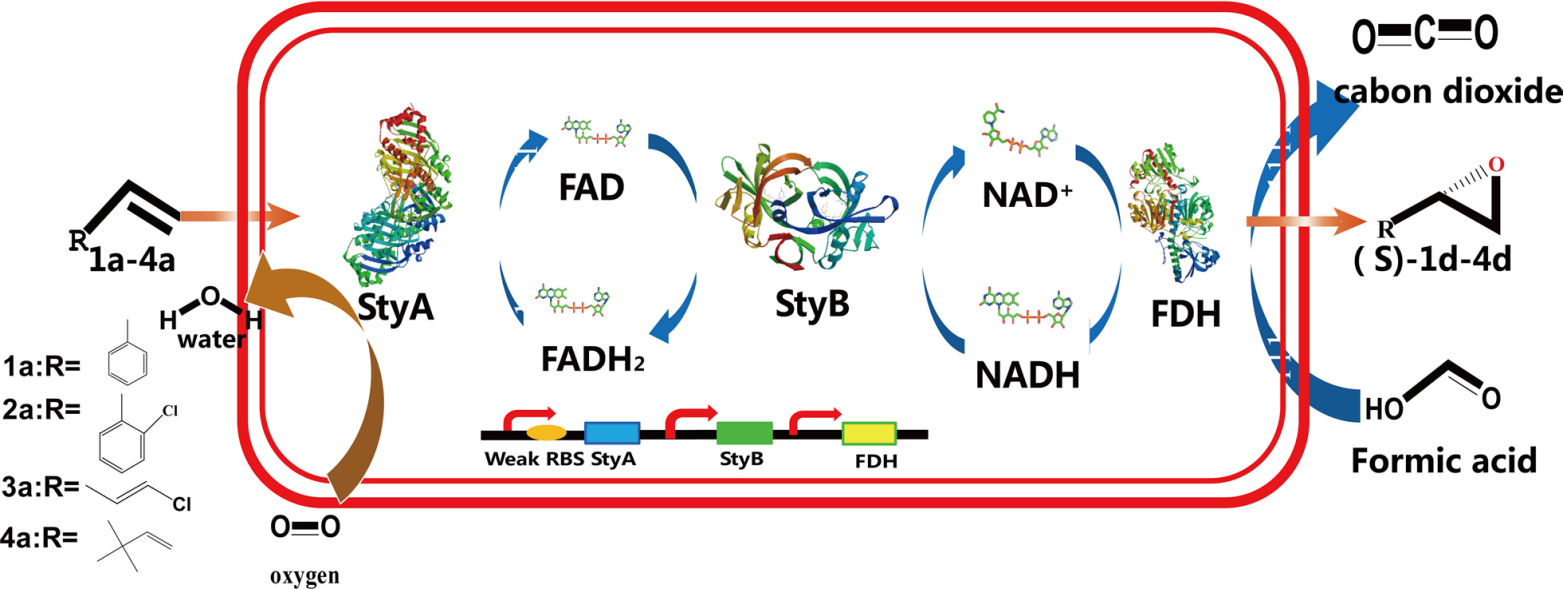
Scheme of the tunable multi enzyme coordinated biocatalytic system with cofactor regeneration for the one-pot conversion of olefins and aromatic compounds to (*S*)-epoxide using recombinant *Pseudomonas putida* KT2440

Based on this screening method, a valuable protocol was provided combining random mutagenesis, site-directed mutagenesis and site-saturation mutagenesis. To discuss the results of this mutation studies, the X-ray crystal structure of the SMO from *Pseudomonas putida* S12 (PDB ID: 3IHM) [23] was used to provide insight into the putative substrate and flavin-binding pockets as well as to model the cofactor FAD docking into the putative active cavity of the SMO from *Pseudomonas* sp. SN1. Afterwards, the expression of the native SMO as well as the mutants in *E. coli* and *Pseudomonas* was investigated as shown in Fig. 1. Finally, a hexadecane/buffer biphasic system was identified as most-promising two-phase system and used to apply these enzymes obtained in whole cells for an application to gain chiral epoxides.

## Results and discussion

### Screening for high enzyme activity mutants from epPCR library

The *styAB* gene (Gene ID: DQ177365.1) encoding for SMO from *Pseudomonas putida* SN1 CGMCC1.2309 was used as a template. The enzyme activity of the SMO derived from *Pseudomonas putida* SN1 was low, with a particularly poor thermostability [24]. In order to improve the SMO activity and thermostability of the enzyme expressed in *E. coli* as well as to attenuate its degree of inactivation in the reaction, a new approach was developed in 96-deep well plates to screen high enzyme activity variants based on color rendering between the epoxide and 4-nitrobenzyl pyridine (4-NBP) from a random mutagenesis library containing more than 2000 SMO mutants [19]. Approximately 56 positive clones with improved color formation on the 96-well plates were identified. However, there were only two mutants with confirmed improved catalytic activity (121% and 195%) compared to the wild type enzyme. At this stage, the mutant with the highest enzyme activity was selected for further optimization. This mutant contained three substitutions D305V, V53G and S189I, defined as A (D305V/V53G/S189I). The new variant with three-site mutations was used as the template for the second round of epPCR. Screening of more than 1000 colonies yielded no positive colonies with improved enzyme activity when compared to those from the first round of epPCR.

**Fig. 2:**
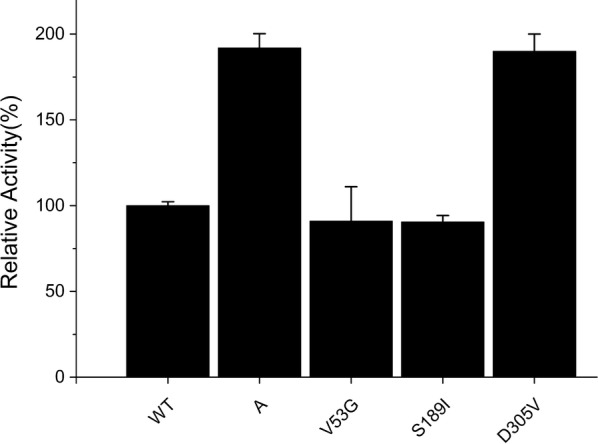
Biotransformation of styrene by the wild type and the mutations. The whole cell biotransformation with the recombinant cells of BL21/pET-28a-*styAB*-*fdh* and its variants were carried out in 50 mL flasks with 10 mL of the KP buffer (200 mM, pH 8.0) containing 26 mM of substrate styrene and 1.0 g cell (dry cell weight) with addition of 50% (v/v) hexadecane at 30 °C and 220 rpm on a rotatory shaker for 15 min. The organic phases were combined, dried with anhydrous sodium sulfate, and subjected to the reverse phase HPLC analysis on a Luna C_18_ column (flow rate: 0.8 mL/min, methanol–water mixture at a ratio of 75:25). Activities were normalized as percentages of the activity of the wild type. 100% corresponds to an initial activity of 72 ± 10 U/g CDW. The variant of A depicts mutation of V53G/S189I/D305V derived by site-directed mutagenesis of the epPCR library. The mutation of V53G, S189I and D305V were derived by site-directed mutagenesis. All assays were performed in triplicate and the standard deviations of the biological replicates are represented by error bars

Site-directed mutation on the three substitutions D305V, V53G and S189I was then carried out in order to verify which substitution had direct effect on enzyme activity. By constructing three single mutations, we found that only the substitution of D305V had significantly enhanced the enzymatic activity, and the other two substitutions of V53G, S189I did not show any significant increase in enzyme activity compared to the wild type (Fig. 2).

### Site-saturation mutagenesis at residue D305 and discussion of the results by structural docking analysis

**Fig. 3:**
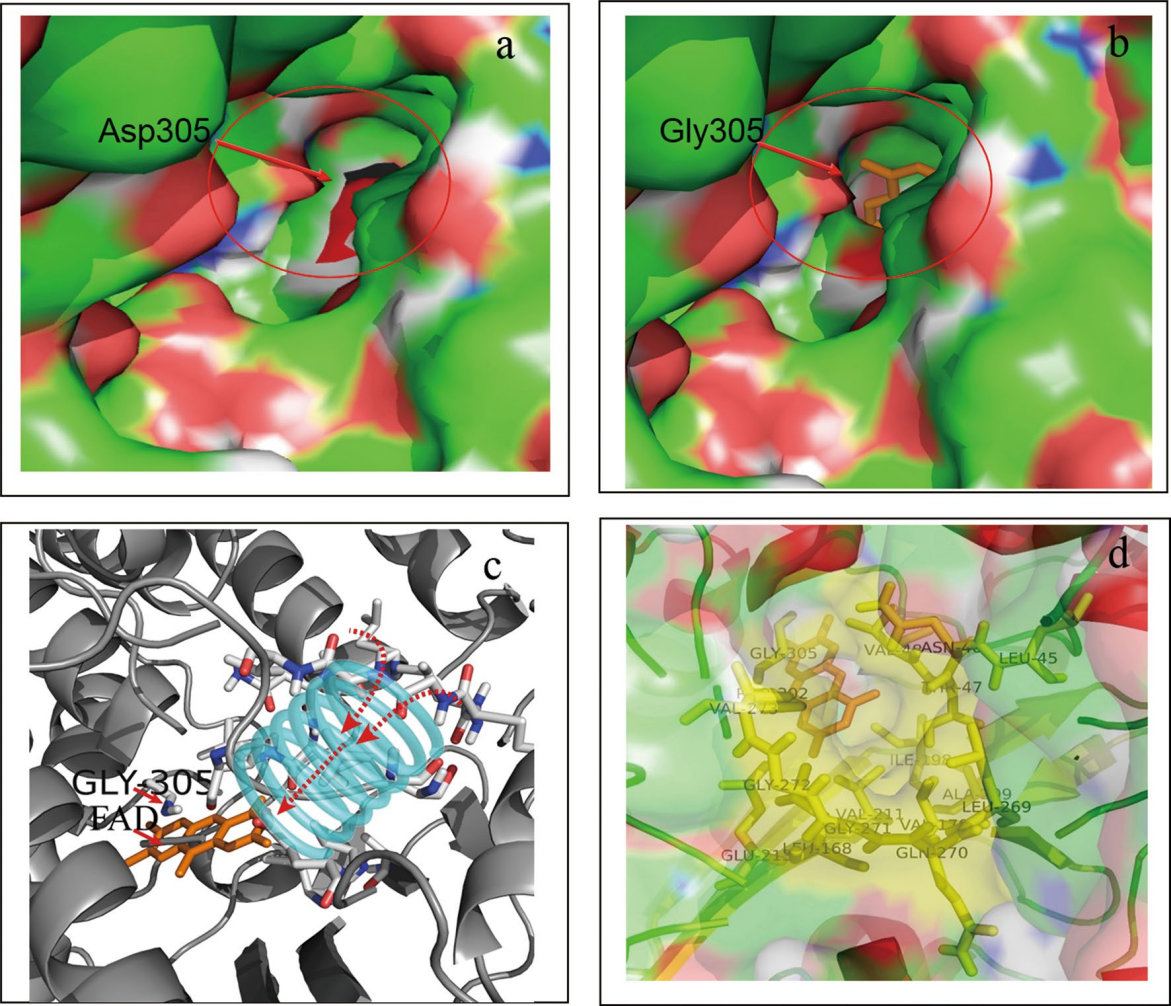
The orientation of FAD docked into the putative active site of SMO (PDB ID: 3IHM). **a** The structure of wild-type SMO (Residue D305G is shown in red of the circle). **b** The structure of the D305G mutant. **c** The channel that interacts with FAD. **d** The channel of residues in the internal structure. The positions of residues predicted to interact with FAD are shown in yellow (the residues Leu45, Val48, Val170, Val211, Leu269, Glu271, Glu272, Glu271, Glu272, Glu213, and Pro302). FAD is shown in orange by the stick mode. The figure was generated using Autodock 4.0 and displayed by Pymol. The number of Autodock 4GA runs was increased from 20 to 40, the docking grids were set as 20 × 22 × 22 Å for styrene and 27 × 30 × 28 Å for flavine adenine dinucleotide

Since the catalytic activity of SMOs is highly influenced by FAD-binding sites, the mutation D305 attracted our interest. A 96-clone library was therefore used to screen all possible SMO mutants at position 305 expressed in *E. coli*. Results indicated that two additional mutants possessed better specific activity than the original mutants from epPCR, with mutant D305G particularly having the highest activity, followed by D305A and D305V (Fig. 2). Structural analysis showed that the side chains at residue 305 dramatically influenced the enzymatic activity of the SMO. Based on the putative FAD-binding channel of SMO, the cofactor FAD molecule was docked into the protein structure using Autodock 4.0 [25]. The returned 20 results were analyzed by MGL tools 1.5.4 and the structure with the highest binding energy score was chosen [26]. As shown in Fig. 3a, b, the amino acid residue D305 is located at the entry of the FAD-binding site and probably plays a very crucial role in catalytic activity [27]. Indeed, it was observed that the residue D305 side chain in the wild type was relatively large and thus hindered access of FAD into the tunnel (indicated as the red region in Fig. 3a), while the side chain of the positive mutant D305G is smaller than that of the wild type and, hence, easily allowed FAD docking into the cavity (Fig. 3b). Flavins are key cofactors in the reductive activation and transfer of oxygen atoms to organic substrates in the biosynthesis of olefins as well as aromatic compounds [28, 29]. As shown in Fig. 3c, the amino acid residue D305G was adjacent to the cofactor FAD. It is well known that residues which are adjacent to the substrate or coenzyme act an important role in catalytic activity [30–32]. As shown in Fig. 3d, it was observed that the amino acid residues of Fig. 3c are the internal structure of the Fig. 3b. Compared with the Asp, the substitution by Val removed the carboxyl acid moiety, however steric hindrance still existed. In summary, results indicated that this structural modification (D305G) decreased the steric hindrance with its missing side chain, making it easier for FAD to access the ligand tunnel with additional space. It can be strongly supposed that these aspects led to an increased catalytic activity with 3.5 times higher *k*_cat_/*K*_m_ values than that of wild type.

**Fig. 4:**
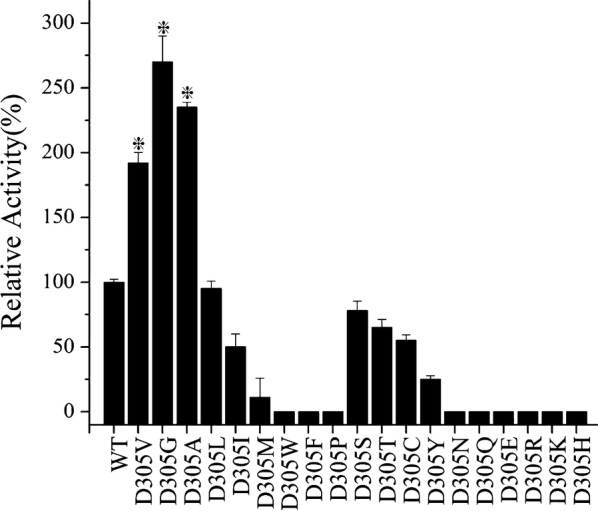
Biotransformation of styrene by the wild type and the site-saturation mutations at D305. The whole cell biotransformation was conducted by the recombinant cells of BL21/pET-28a-*styAB*_D305X_-*fdh* and its wild type (X = V, A, G). The reaction was carried out in 50 mL flasks with 10 mL of KP buffer (200 mM, pH 8.0) containing 26 mM of substrate styrene and 1.0 g cell of dry weight with addition of 50% (v/v) hexadecane at 30 °C and 220 rpm on a rotatory shaker for 15 min. After incubation, the product was withdrawn and subjected to the reverse phase HPLC analysis. The reaction mixture was used to determine specific epoxidation activities. The enzyme activity of wild type was set to 100%, corresponding to the initial activity of 72 ± 10 U/g CDW. All assays were performed in triplicate and the standard deviations of the biological replicates are represented by error bars

Furthermore, it is well recognized that hydrophobicity of the active site tunnel is an important factor in determining the enzymatic specificity and stability [33]. Structural analysis of the large cavity forming the FAD binding site and the ligand tunnel showed that these structures are completely buried within many hydrophobic residues and only few hydrophilic residues (Fig. 3c, d) which is consistent with the hydrophobic nature of the substrate styrene [23]. Therefore, hydrophobic interactions appeared to be crucial at position 305. Replacement of the Asp residue with Gly or Ala increased the enzyme activity, while the loss of the hydrophobic side chain resulted in significantly decreased activity for D305S, D305T as well as in the case of the wild type itself (Fig. 4).

### Expression and purification of wild type and mutant enzymes

**Fig. 5:**
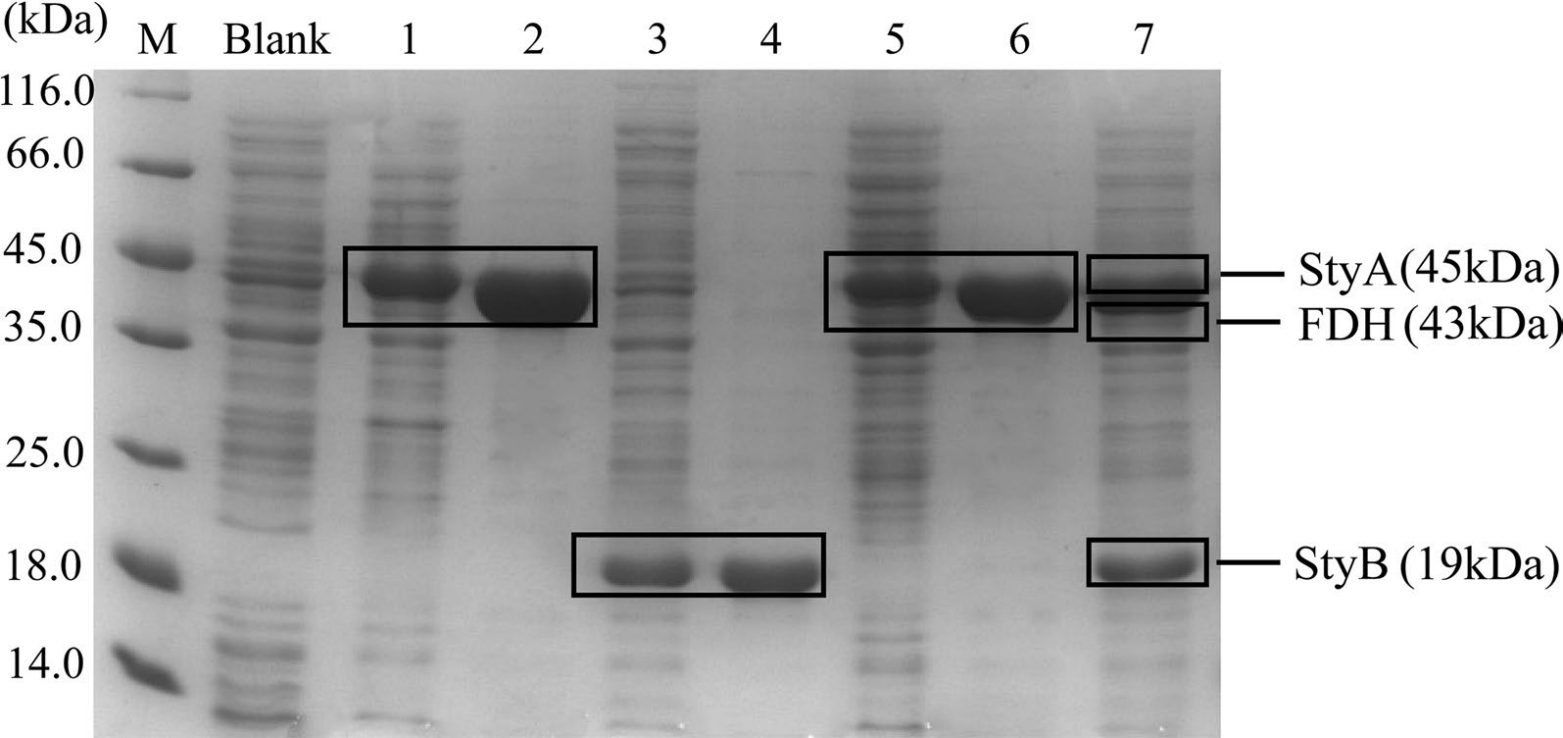
SDS-PAGE analysis of the overexpression of StyA, StyB and FDH in different hosts. The following samples and markers are shown: M, protein marker; Blank, whole cell protein of control *E. coli*; Lane 1, whole cell protein of *E. coli*-pET28a- *styA*; Lane 2, purified proteins of *E. coli*-pET28a-*styA*; Lane 3, whole cell protein of *E. coli*-pET28a-*styB*; Lane 4, purified proteins of *E. coli*-pET28a-*styB*; Lane 5, whole cell protein of *E. coli*-pET28a-*fdh*; Lane 6, purified proteins of *E. coli*-pET28a-*fdh*; Lane 7, whole cell protein of *Pseudomonas putida* KT2440/pJB861-*styAB*-*fdh*

The wild type and mutants were all expressed in *E. coli* BL21 and induced with IPTG as mentioned in “Materials and methods”. Three clear bands with a molecular weight of about 45 kDa for StyA, 19 kDa for StyB and 43 kDa for formate dehydrogenase (FDH: EC 1.2.1.2), respectively, were observed after the 6× His tagged purified protein was analyzed by 15% SDS-PAGE. The NAD^+^-dependent FDH was expressed in this study for NADH regeneration in order to avoid the need for addition of external NADH which is required for the efficient conversion of the substrate by SMO [34]. On the other hand, this homodimeric enzyme could catalyze the oxidation of formate ion to carbon dioxide. Because of that, by-product formation could be minimized. The *styB* gene was inserted into the pET-28a plasmid and expressed in *E. coli* BL21 (DE3) as C-terminal His6-tagged protein; otherwise most of the StyB proteins would be expressed in the form of insoluble inclusion bodies. It is well known that there is a lengthy sequence in front of the multiple cloning site of pET-28a, including the N-terminal His-tags, which may cause incorrect folding of the protein. Therefore, we removed the N-terminal His-tags and changed it to the C-terminus. In order to realize that, the PCR products were purified and digested with *Nco*I and *Hin*dIII at 37 °C for 45 min, then ligated into the pET-28a vector, which was also digested with the same endonucleases. The products were then transformed into *E. coli* BL21 (DE3), plated on Luria–Bertani (LB) agar. These changes improved *styB* gene expression obtaining more than 60% of StyB as soluble protein (Fig. 5). The purified StyA, StyB and FDH were used for testing the enzyme properties. Certainly, there were also some other useful ideas for recovering the StyB protein by resolubilization from inclusion bodies [35]. Moreover, the solubility or purification results were all the same among the mutations. Finally, the recombinant plasmids pJB861/*styAB*_D305X_-*fdh* were constructed and transformed it into competent cells of *Pseudomonas putida* KT2440 by electroporation (X = V, A, G), as shown in Fig. 5. The recombinant strains of *Pseudomonas putida* KT2440/pJB861/*styAB*_D305X_-*fdh* (X = V, A, G) were then used for the whole cell biotransformation of the native substrate styrene and other short chain compounds such as 3-allyl chloride.

### Enzyme characterization of SMO

**Fig. 6:**
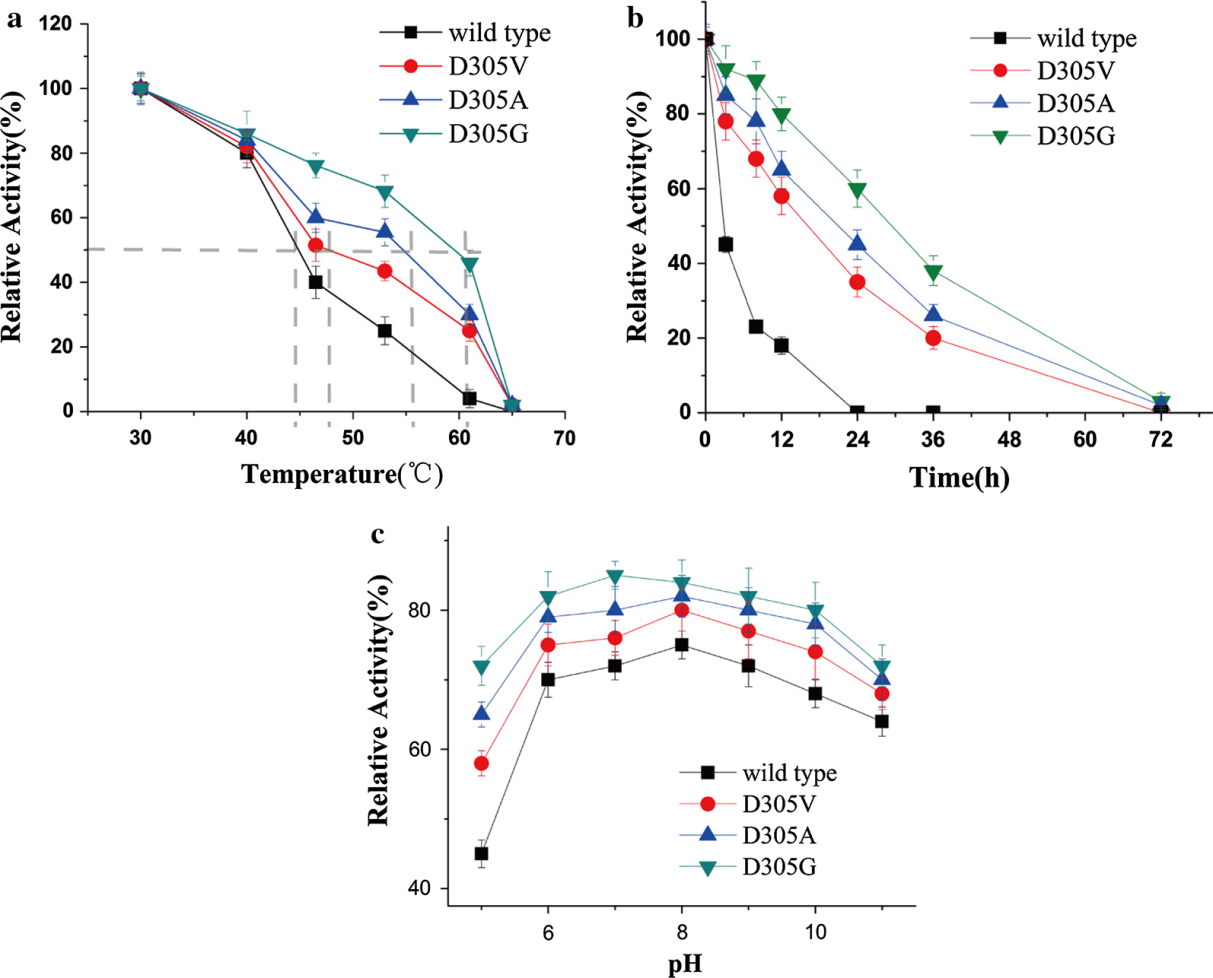
Enzymatic stability for wild type SMO and its variants in *E. coli* expression host. Thermal stabilities of the enzymes were carried out by purified enzyme incubation in KP (potassium phosphate) buffer (200 mM, pH 8.0) for 12 h at a range of temperatures from 30 to 65 °C. Afterwards, the residual activity of the pre-incubated SMOs was tested in the following reaction system: 0.8 U/mL purified SMOA, 1.6 U/mL of purified SMOB, 1.7 U/mL formate dehydrogenase, 0.2 M sodium formate, 0.3 mM NADH, 1 mM NAD^+^, 0.05 mM FAD, and 200 mM styrene (from a 200-fold stock in ethanol). And the pH stability of the wild type and variants was determined by purified enzyme incubation at 30 °C for 12 h in the following buffer system: 0.05 M acetate buffer (pH 3.0–6.0), 0.05 M phosphate buffer (pH 6.0–8.0), and 0.05 M glycine–NaOH buffer (pH 8.0–11.0). After incubation, their residual enzyme activities were measured at 30 °C in KP buffer (200 mM, pH 8.0). **a** Enzyme inactivation assay at different temperatures for 12 h. **b** Time courses of thermal inactivation at 60 °C. **c** Enzyme inactivation assay at different pH for 12 h. The initial activity before incubation was set to 100%, 100%-value corresponds to an initial activity of 5.6 ± 0.21 U/mg. All assays were performed in triplicate and the standard deviations of the biological replicates are represented by error bars

Temperature and pH are very important parameters that influence enzyme activity and stability. The purified enzyme samples (wild type and mutant) expressed in *E. coli*, were used to determine the effect of temperature, pH, and thermostability on the SMO enzyme. In this assay, 200 mM potassium phosphate buffer containing the substrate and co-substrate were used to suspend the purified protein. 2 mL of the mixture were incubated in 10 mL flasks on the rotary shaker for 1 h. To identify the optimum temperature for styrene conversion, the temperature during the biotransformation of styrene with the wild type SMO was adjusted from 5 to 45 °C and the maximum activity was finally observed at 30 °C (Additional file 1: Figure S3a). The enzyme activity in presence of these temperatures significantly dropped at higher reaction temperatures above 35 °C (Additional file 1: Figure S3a). Based on that, a temperature of 30 °C was used for all subsequent activity measurements. In the case of thermostability (= 12-h-pre-incubation of native SMO and variants at different temperatures before styrene addition/activity assay; see Fig. 6a), there was no significant difference between the wild type and the variants below 40 °C. However, the substitutions D305V, D305A and D305G resulted in a large improvement in thermostability with the Tm values of 47.5 °C, 54.8 °C, 59.8 °C, respectively, which were higher than that of the wild type SMO (44.2 °C). As shown in Fig. 6b, the variants D305V, D305A, D305G were more stable than the wild type at 60 °C. The half-lives of thermal inactivation (t_1/2_) at 60 °C for D305V D305A and D305G variants were 24 h, 23 h and 28.9 h respectively, which correspond to a 7.2-, 7.5- and 9.0-fold increase to that of wild type (3.2 h). According to the experiments above, the principle pH optimum for the transformation was determined using the wild type SMO during a preliminary experiment. The optimum reaction pH of the enzyme was 8.0 (Additional file 1: Figure S3b). Based on that, a pH of 8.0 was used for all subsequent activity measurements. Afterwards, further pH-stability experiments with the mutants D305V, D305A and D305G were performed by a 12-h-pre-incubation of the enzymes at different pH values in comparison to the wild type (see Fig. 6c). The subsequent activity measurements revealed that the mutants showed a higher stability under acidic conditions pH (pH 5.0) compared to the wild type. As shown in Fig. 6c, the wild type showed poor acid resistance retaining only 45% residual activity left after 12 h incubation at pH 5.0. However, under alkaline conditions (pH 10.0), all the mutants and wild type showed residual activity of about 70% after 12 h incubation. These results indicated that an improvement of the hydrophobicity of the substrate entrance tunnel contributed not only the improved enzymatic activity, but also the enhanced stability of the enzyme.

### Kinetic analysis of the variants and the wild type enzyme towards the substrate styrene

Kinetic parameters of the purified wild type SMO and the mutants in *E. coli* expression hosts were measured using different concentrations of the substrate styrene ranging from 0.5 to 16 mM. The reactions were carried out in 2 mL volumes and the apparent *K*_m_ and *k*_cat_ values were determined from Michaelis–Menten kinetic plots by the software origin 8.5. As shown in Table 1, the mutations D305V, D305A, and D305G demonstrated a better catalytic efficiency with the substrate styrene compared to the wild type enzyme. The variant D305G showed nearly 3.5 times higher *k*_cat_/*K*_m_ values than the wild type. All the three substitutions were hydrophobic amino acids with shorter side chains at the entrance of the tunnel, which confirmed the importance of hydrophobicity for the ligand entrance tunnel.

**Table 1:** The kinetic parameters of mutants screened from site-saturation mutagenesis at position 305 compared to the wild type

Mutations	*K*_m_ (mM)	*k*_cat_ (s^−1^)	*k*_cat_/*K*_m_ (mM^−1^ s^−1^)
Wild type	0.85 ± 0.12	1.08 ± 0.03	1.27 ± 0.15
D305V	0.54 ± 0.08	1.43 ± 0.03	2.65 ± 0.17
D305A	0.46 ± 0.11	1.53 ± 0.02	3.33 ± 0.21
D305G	0.36 ± 0.14	1.70 ± 0.02	4.72 ± 0.33

The reaction was carried out in 2 mL volumes containing 0.2 M KP (potassium phosphate) buffer (pH 8.0), 0.8 U/mL purified SMOA, 1.6 U/mL of purified SMOB, 1.7 U/mL formate dehydrogenase (FDH; EC 1.2.1.2, from *Candida boidinii*), 0.2 M sodium formate, 0.3 mM NADH, 1 mM NAD^+^, 0.05 mM FAD, and varying concentrations of styrene (from a 200-fold stock in ethanol). All enzymes were previously expressed in *E. coli* BL21. The reaction mixture was shaken at 200 rpm and 30 °C for 1 h, and then it was extracted with ether and analyzed with reverse phase HPLC on a Luna C_18_ (4.6 mm × 150 mm) column at a flow rate of 0.8 mL/min

### Screening of water-miscible and water-immiscible organic solvents for the application in a SMO-catalyzed biotransformation

An appropriate biphasic system could be used to improve the biocatalytic efficiency by enhancing the enzyme tolerance to toxic substrate or product [16]. Therefore, biocompatibility of the D305G variant expressed in *Pseudomonas putida* KT2440 was taken into consideration in order to screen the appropriate organic solvents for the enzyme reaction [36]. Four commonly used water-miscible organic solvents were then selected to determine their effects on SMO at different concentrations (shown in Additional file 2: Figure S2a). The results indicated that 10% (v/v) concentration of the four solvents (ethanol, isoamylalcohol, DMSO, isopropanol) had a minimal effect on SMO activity as the enzyme retained more than 87% of its initial activity. However, when the concentration of the organic solvent was increased, the enzyme activity of the SMO was significantly reduced. When 30% of the water-miscible organic solvent was added, the SMO retained 0–12% of the initial enzyme activity. This phenomenon was probably due to the fact that such polar solvents tend to denature proteins and thus led to the loss of the enzyme activity. The least toxic effect on the SMO activity was observed when 15% of isoamylalcohol was used in the reaction with the enzyme retaining 90% of the initial activity in this solvent.

**Fig. 7:**
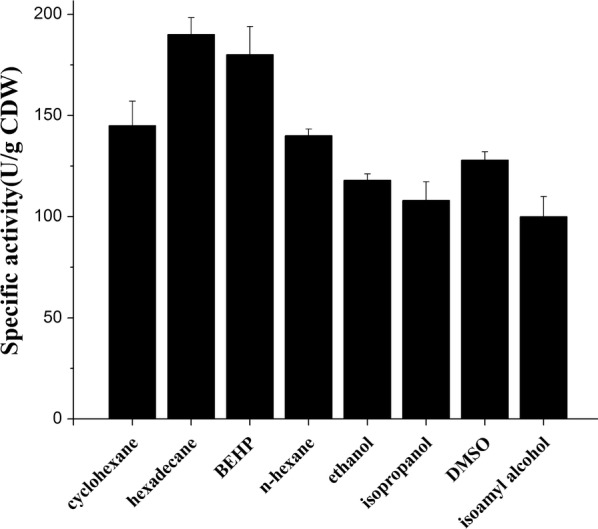
Biotransformation of styrene to (*S*)-styrene oxide by whole cells of recombinant *Pseudomonas putida* KT2440 in different solvents. The whole cell biotransformation was conducted by *Pseudomonas putida* KT2440/pJB861-*styAB*_D305G_-*fdh* toward styrene. The reaction was carried out in 50 mL flasks with 10 mL of KP buffer (200 mM, pH 8.0) containing 26 mM of the substrate styrene and biomass with a cell dry weight of 1.0 g, incubated at 30 °C for 8 h. And the reaction system was substituted with different water-miscible (10%, v/v) and water-immiscible (1:1, v/v) solvents. The product was withdrawn periodically and analyzed by reverse phase HPLC on a Luna C_18_ column at a flow rate of 0.8 mL/min under a methanol–water mixture at a ratio of 75:25. The reaction mixture after 15 min was used to determine specific epoxidation activities. All assays were performed in triplicate; each column represents the mean of triplicate assays

Another approach explored to protect this enzyme against inhibition was addition of a water-immiscible organic solvent to the reaction system (organic solvent/buffer, 1:1). The organic solvent reduces the contact between the enzyme and the substrate, because the biocatalyst remains in the aqueous phase while the substrate accumulates in the organic phase. Thus, the substrate toxicity as well as the product inhibition are commonly reduced, and the enzyme activity is enhanced. Among the eight solvents tested (see Additional file 2: Figure S2b), the SMO was very stable in the water-immiscible organic solvents containing hexadecane, *n*-hexane, cyclohexane and bis-(2-ethylhexyl) phthalate (BEHP) retaining more than 75% of its initial enzyme activity after 8 h incubation and the SMO showed the lowest stability in dichloromethane (5%), followed by toluene (14.5%), trichloromethane (45%), ethyl acetate (58.5%). Ultimately, the following eight biphasic systems were constructed with good biocompatibility to the SMO: 10% DMSO/buffer, 10% ethanol/buffer, 15% isoamylalcohol/buffer, 10% isopropanol/buffer, cyclohexane/buffer (1:1), BEHP/buffer (1:1), hexadecane/buffer (1:1), and *n*-hexane/buffer (1:1). The application of hexadecane was the most effective for (*S*)-styrene oxide production using the recombinant cells of *Pseudomonas putida* KT2440/pJB861-*styAB*_D305G_-*fdh*, reaching a maximum activity of 190 ± 13 U/g CDW (see Fig. 7), which was 2.4 to 3.5 times higher than that of the other SMOs measured under similar conditions, such as that from *Pseudomonas* sp. VLB120 (79 ± 5 U/g CDW) and *P. putida* SN1 (55 ± 5 U/g CDW) [6, 24]. In addition, the total amounts of 20.5 mM (*S*)-styrene oxide were reached in 8 h (as shown in Additional file 3: Figure S6). The average product conversion rate finally reached 2.56 mM/h. This corresponds to a 2.7-fold higher rate than the rate of another SMO from *Pseudomonas putida* CA-3 which was measured under similar conditions during a previous study (0.95 mM/h) [4]. The high yield obtained in this study could be attributed to the effectiveness of the hexadecane system in subduing substrate toxicity and product inhibition on the biocatalytic system. Based on the results above, hexadecane was selected as the best organic solvent for constructing a biphasic system.

### Expression of SMO in recombinant *Pseudomonas putida* KT2440 and application for epoxidation

The expression of SMO in *Pseudomonas putid*a KT2440 as an alternative host was explored in this study, because this strain is an archetype environmental microbe that has been genetically studied and previously reported as an excellent host for heterologous gene expression [37]. Since the *styAB* gene was derived from the *Pseudomonas putida,* a *Pseudomonas* expression host seems to be a promising choice for efficient SMO expression. Furthermore, *Pseudomonas putida* strains produced rhamnolipids [38]. Such rhamnolipids are anionic biosurfactants that can help to degrade polycyclic aromatic hydrocarbon compounds and emulsify alkanes [39, 40]. They also have a strong ability to reduce surface tension to approximately 30 mN/m [41], thus enhancing interaction between enzyme and the substrate during biotransformation. In addition, *Pseudomonas putida* KT2440 is a model environmental microbial strain, which is the most clear and genetically studied *Pseudomonas putida* strain [42]. It is the preferred host for gene cloning and expression because of its simple and clear genetic operating system. The improved highly efficient electroporation method of *P. putida* KT2440 has already been established [43]. Based on all of these facts, the recombinant strains *Pseudomonas putida* KT2440/pJB861-*styAB*_D305X_-*fdh* (SMO mutants, X = V, A, G) and *Pseudomonas putida* KT2440/pJB861-*styAB*-*fdh* (wild type SMO) were constructed to catalyze the transformation of the substrates styrene, 2-chlorostyrene (aromatic compounds**)** and other important short chain compounds (3,3-dimethyl-1-butene and 3-allyl chloride). The results showed that the conversion rate of *Pseudomonas putida* KT2440/pJB861-*styAB*_D305G_-*fdh* for the substrates was higher than that in wild type (Table 2).

**Table 2:** Substrate conversion and enantiomeric excess for the bioepoxidation of styrene, 3,3-dimethyl-1-butene, 3-allyl chloride and 2-chlorostyrene using the recombinant cells of *Pseudomonas putida* KT2440 (wild type and mutants) of SMO

Mutant								
	Conversion (%)	ee (%)	Conversion (%)	ee (%)	Conversion (%)	ee (%)	Conversion (%)	ee (%)
Wild type	85	> 99 (*S,S*)	58	71 (*S,S*)	42	52 (*S,S*)	76	> 99 (*S,S*)
D305A	93	> 99 (*S,S*)	67	70 (*S,S*)	51	48 (*S,S*)	86	> 99 (*S,S*)
D305V	91	> 99 (*S,S*)	62	72 (*S,S*)	48	50 (*S,S*)	81	> 99 (*S,S*)
D305G	95	> 99 (*S,S*)	71	70 (*S,S*)	54	53 (*S,S*)	97	> 99 (*S,S*)

The culture conditions are as follows: the single colonies were grown over 24 h at 30 °C in EM medium containing kanamycin (50 μg/mL). One milliliter of the culture was then inoculated into 100 mL of EM medium, then cultured at 30 °C for 24 h to induce protein expression. The cells were harvested by centrifuging at 8000×*g* for 8 min at 4 °C. The whole cell biotransformation with *Pseudomonas putida* KT2440/pJB861-*styAB*_D305X_-*fdh* (SMO mutants, X = V, A, G) and *Pseudomonas putida* KT2440/pJB861-*styAB*-*fdh* (wild type SMO) were carried out in 50 mL flasks containing 10 mL of 200 mM KP buffer (pH 8.0) with 26 mM of one substrate, dry cell weight of 1.0 g, and 50% (v/v) hexadecane, incubated at 30 °C and 220 rpm on a rotatory shaker for 8 h. The product formation was determined by HPLC and the conversion was determined for the initial 30 min, as mentioned in “Materials and methods”

## Conclusions

In conclusion, the enzymatic activity of SMO from *Pseudomonas* sp. SN1 was enhanced by screening and engineering some crucial residues adjacent to its flavine adenine dinucleotide (FAD) binding pocket. A new method for screening high-activity mutants from a random mutant library was designed. Using this method, a three-site mutant with 1.9-fold increased enzymatic activity than that of wild type was obtained. The variant with D305V mutation was the most active one after analyzing the results of site-directed mutagenesis yielding 1.9-fold higher activities than the wild type. Moreover, the docking results showed that the residue D305 was located at the FAD-binding cavity and its large side chain blocked FAD from accessing the cavity and thus played a key role in coenzyme interaction with the active site. Site-saturation mutagenesis at this position 305 resulted in two more mutants (D305A and D305G) with better specific activities (2.3- and 2.7-times, respectively), and higher half-live times during thermal inactivation compared to the wild type. The engineered SMO was also successfully expressed with high yields in *Pseudomonas putida* KT2440 and *E. coli*. Finally, aromatic as well as other substrates were transformed with an established hexadecane/buffer biphasic system, and the results indicated decreased substrate toxicity and product inhibition for the mutants. Finally, the average product conversion rate reached 2.56 mM/h using the mutant D305G, which was 2.7 times higher than that of *Pseudomonas putida* CA-3 (0.95 mM/h) [4].

## Materials and methods

### Strains, plasmids and chemicals

The *styAB* gene (Gene ID: DQ177365.1) encoding SMO from *Pseudomonas putida* SN1 CGMCC1.2309 was used as a template. Moreover, the two bacterial strains *Escherichia coli* BL21 and *Pseudomonas putida* KT2440 were used as hosts for gene (*styAB*) expression, respectively. The plasmid pET-28a (+) purchased from Invitrogen (Carlsbad, CA) was used for the enzyme expression and mutagenesis studies. The substrates styrene, 3,3-dimethyl-1-butene, 3-allyl chloride and 2-chlorostyrene were purchased from J&KChemical Ltd. (Shanghai, China). The styrene oxide, epoxy 3,3-dimethyl-1-butene, epoxy chloropropene and 2-chlorostyrene oxide was bought from Sigma-Aldrich Chemical Co. Inc. (Shanghai, China). Prime STAR DNA polymerase was sourced from TaKaRa BioCo. (Dalian, China), 2x Phanta Max Master Mix was bought form Vazyme (Nanjing, China) and other reagents of high quality were obtained from general suppliers in Wuxi. The bacterial strains and plasmids used in this study are listed in Additional file 4: Table S1 whereas all the primers are summarized in Table 3.

**Table 3:** Primers used in this work

Primers	Sequences 5′–3′
N46S F	TGGTCTGCGTTTACTG**AGC**ACAGTTGCCCACAATG
V48G F	GCGTTTACTGAATACA**GGT**GCCCACAATGCCGTGA
V48Q F	GCGTTTACTGAATACA**CAG**GCCCACAATGCCGTGA
M186L F	GATTCGCGCAGTGACA**CTG**AGCTTCAGCCCGGGTC
M186G F	GATTCGCGCAGTGACA**GGT**AGCTTCAGCCCGGGTC
L269G F	CAGTTCTTTAGACATC**GGT**CAAGGTGGCGTTGTGC
L269V F	CAGTTCTTTAGACATC**GTT**CAAGGTGGCGTTGTGC
D305A F	CGTTGATCCGGTTCTG**GCC**CAAGGTGCCAATATGG
D305X F	CGTTGATCCGGTTCTG***NNN***CAAGGTGCCAATATGG
R	GCCTTACTGGTTAGCAGAATG

The bold type shows the site of the variations. Saturated mutation sites were substituted by bold and italic ***NNN*** (N = A/G/C/T). In addition, all the site of variations share the same downstream primer

### Construction of the mutant library

The mutagenesis library was constructed by error-prone PCR (ep-PCR) according to the manufacturer’s instructions of Gene Morph Random Mutagenesis kit [44]. We moderated the frequency of mutation to 2–4 mutations/kb by adjusting the concentration of the initial template during the PCR reaction. The primers 5′-ATGAAGAAACGCATTGGCATTGT-3′ and 5′-TGCTGCAATGGTCGGTGC-3′ were used to construct epPCR libraries of SMO. The recombinant plasmid pET-28a-*styA* encoding wild type styrene monooxygenase was used as the template for the first round of epPCR and the most active mutant from the first round of epPCR was used as template for the second round of epPCR.

The fragments of amplified PCR products were purified and digested by *Nco*I and *Hin*dIII at 37 °C for 45 min and then ligated into the vector pET28a, which was also digested with the same endonucleases. Then the products were transformed into *E. coli* BL21 (DE3), plated on Luria–Bertani (LB) agar containing 50 μg/mL kanamycin then cultured at 37 °C for 12 h.

### Screening for high-activity variants

Single colonies from the mutant library were picked from Luria–Bertani (LB) plates containing kanamycin (50 μg/mL) and incubated at 37 °C in 96-well microplates overnight. The overnight bacterial culture (100 μL) was then transferred to fresh 96-deep well plates containing LB liquid medium incubated at 37 °C and 160 rpm for 2 h, induced for enzyme expression with 1 mM IPTG for 2 h then cultured for 12 h. The cells were harvested by centrifugation at 2260×*g* for 10 min. The supernatant was removed from the 96-deep well plates, then the *E. coli* cells were suspended to a cell density of 1.0 g cdw/L in KP (potassium phosphate) buffer (0.2 M pH 8.0) containing glucose (2%, w/v) and 40 μL of a substrate stock solution (0.2 M in ethanol) in a 2 mL system. The reaction mixture was incubated at 30 °C for 30 min with 200 rpm shaking [2].

In a stoppered test tube, 200 μL of the reaction mixture was pipetted into a mixed solution containing 800 μL of buffer A (buffer A is a mixture of 50 mM K_2_HPO_4_-KH_2_PO_4_, pH 7.0) and 400 μL of 10 mM 4-NBP (4-NBP is equimolded with an equal volume of ethylene glycol and acetone; the concentration of the mother liquor is 100 mM) and then reacted at 80 °C for 10 min [45]. Afterwards, the mixture was immediately cooled down by ice bathe for 5 min. In addition, 400 μL of 50% (v/v) trimethylamine-acetone buffer were added into the mixture and the total solution turned blue. The samples were subsequently measured by spectrophotometer at an absorbance–wavelength of 565 nm [46]. The higher specific activity mutants were selected for further studies. *E. coli* BL21/pET28a-*styAB* (wild type) was used as a control.

### Site-directed and site-saturation mutagenesis

Site-directed and site-saturation mutagenesis of the *styAB* gene at D305 were carried out using whole-plasmid two-step PCR method [47]. The mutation primers used for this are listed in Table 3. Site-saturation mutagenesis at position D305 was accomplished by the degenerate codon of *NNN* (N represents A, T, G or C). In addition, the improved fragments of amplified PCR products were purified and digested by *Nco*I and *Hin*dIII at 37 °C for 45 min and then ligated into the vector pET28a, which was also digested with the same endonucleases. The recombinant plasmids were finally transformed into *E. coli* BL21 (DE3) and sequenced by Sangon Biotech to confirm successful mutations.

### Structure simulation and molecular docking studies

The X-ray crystal structure of the oxygenase subunit of *P. putida* S12 StyA was obtained from the PDB database (PDB ID: 3IHM). The *P. putida* S12 StyA has 90% amino acid sequence similarity to that from *Pseudomonas putida strain* SN1 (Additional file 5: Figure S1). The structure models of the wild type SMO were constructed by homology modeling and downloaded from SWISS-MODEL Workspace (http://swissmodel.expasy.org/) [48, 49]. The 3D structure of the styrene ligand and the cofactor flavine adenine dinucleotide (FAD) were downloaded from http://www.chemspider.com/website and prior to docking, all water molecules were removed and non-polar hydrogen atoms added using MGLTools 1.5.4 [50]. AutoDock 4.2 software (Scripps Institute, California, USA) was used for docking. The number of AutoDock 4GA runs was increased from 20 to 40, the docking grids were set as 20 × 22 × 22 Å for styrene and 27 × 30 × 28 Å for flavine adenine dinucleotide (FAD) [50]. The 10 independent runs of the ligand-receptor complex from AutoDock 4.2 were calculated by the energy interaction value using MGLTools 1.5.4 and the best docking pose was chosen and visualized by Pymol.

### Expression and purification of recombinant proteins from *E. coli*

The single colonies were grown overnight at 37 °C in LB media containing 50 μg/mL kanamycin. One milliliter of overnight culture was then inoculated into 100 mL of Luria–Bertani (LB) medium also containing 50 μg/mL kanamycin, then cultured at 37 °C until the OD_600_ concentration reached 0.4. Isopropyl-d-1-thiogalactopyranoside(IPTG)was added to a final concentration of 0.5 mM in medium to induce protein expression. Afterwards, the cells were cultured at 20 °C for 18 h. The cultures were subsequently harvested by centrifuging at 8000×*g* for 8 min at 4 °C.

Recombinant *E. coli* cells (wet weight) treated with lysozyme were lysed by sonication in buffer A, consisting of 0.1 M potassium phosphate (pH 8.0), 20% glycerol (v/v), 1 mM phenylmethyl sulfonylfluoride (PMSF), 0.5 M potassium chloride, 0.1 mM dithiothreitol (DTT) and 5 mM imidazole [51]. The lysate was centrifuged at 8000×*g* for 20 min, and the supernatant was purified in a 1 mL HisTrapTM HP column on an AKTA purifier system (GE Healthcare, Sweden) with binding buffer (0.02 M Tris–HCl bufer and 0.5 M NaCl, pH 7.4) at a 0.5 mL/min loading rate. The purified protein was eluted at 1 mL/min flow rate against a linear gradient of imidazole concentrations in buffer A ranging from 0 to 0.5 M. Then, the purified enzymes were pooled for SDS-PAGE analysis. A Bradford protein assay kit was used to determined protein concentration [52].

### Enzyme assay and HPLC analytics

The enzyme activities and the kinetic parameters were determined using purified enzymes from *E. coli* by measuring the formation of styrene oxide using HPLC with 2 mL volumes, consisting of 0.2 M KP buffer (pH 8.0), 0.8 U/mL of purified SMOA, 1.6 U/mL of purified SMOB, 1.7 U/mL of purified formate dehydrogenase (FDH: EC 1.2.1.2, from *Candida boidinii*), 0.2 M sodium formate, 0.3 mM NADH, 1 mM NAD^+^, 0.05 mM FAD, and varying concentrations of styrene (from a 200-fold stock in ethanol). The reaction mixture was shaken at 200 rpm and 30 °C for 12 h. Furthermore, the mixture was extracted with ether and analyzed with reverse phase HPLC on a Luna C_18_ (4.6 mm × 150 mm) column at a flow rate of 0.8 mL/min. The mobile phase consisted of a methanol–water mixture at a ratio of 75:25. One unit (U) is defined as the activity that produces 1 μmol of oxide per min.

Specific epoxidation activities were measured using the whole cells and it was calculated as an average activity based on the amount of product formed in given, constant time with the knowledge of the possibility that substrate conversion is not linear to time during the assay period [24, 53]. Experiments were repeated at least three times independently. The recombinant cells of *E. coli* BL21 or *Pseudomonas putida* KT2440 with 1.0 g CDW/L were resuspended in 10 mL KP buffer (200 mM, pH 8.0) and 50% (v/v) hexadecane containing 26 mM (in organic solvent) of one of the following substrates: styrene, 2-chlorostyrene, 3,3-dimethyl-1-butene or 3-allyl chloride (Additional file 6: Figure S5). Afterwards, the cultures were incubated at 30 °C for 8 h at 220 rpm on a rotatory shaker and terminated by extraction with ether. The reaction mixture that lasted for 15 min was used to determine the specific epoxidation activities. The combined organic extracts were dried with anhydrous sodium sulfate and subjected to HPLC analysis. Chemical yields were analyzed by reverse-phase HPLC on a Luna C_18_ column at a flow rate of 0.8 mL/min.

### Determination of the SMO enzyme properties and kinetics assays

The optimum temperature of SMO from the *E. coli* was determined using 200 mM potassium phosphate buffer (pH 8.0) with temperature ranging from 5 to 45 °C. The optimum pH was measured by assaying the enzyme activity at various pH values (0.05 M acetate buffer, pH 3.0–6.0; 0.05 M phosphate buffer, pH 6.0–8.0; 0.05 M glycine–NaOH buffer, pH 8.0–11.0) at 30 °C. In addition, thermal stabilities of the enzymes were carried out by incubation in KP (potassium phosphate) buffer (200 mM, pH 8.0) for 12 h at a range of temperatures from 30 to 65 °C. The residual enzyme activities of the wild type SMO and its variants after incubation were all measured at 30 °C in KP buffer (pH 8.0). Furthermore, the FDH with high stability and activity was used as the coenzyme for SMO. When measuring the residual enzyme activity of SMO, an excess of formate dehydrogenase was added to ensure adequate supply of coenzyme. Moreover, the pH stabilities of the wild type and its variants were determined by purified enzymes incubation at 30 °C for 12 h in different buffers with pH values ranging from 5 to 11. After incubation, their residual activities were measured in KP buffer (0.05 M, pH 8.0) at 30 °C. For the thermal stabilities and pH stabilities, only the SMO was pre-incubated at different temperatures or pH and then the residual enzyme activity was measured at 30 °C and pH 8.0. However, the FDH (EC 1.2.1.2: formate dehydrogenase) was not incubated at different temperature or pH values. The Tm value defined as the temperature, at which half of the initial enzyme activity remained, was determined according to the plots of residual relative activity (%) versus temperature (°C). Activity of wild type SMO and its variants at 30 °C in KP buffer (0.05 M, pH 8.0) without incubation was defined as 100%.

Kinetic parameters of the wild type SMO and variants were determined in KP buffer (0.05 M, pH 8.0) at 30 °C with varied concentration of the substrate styrene (with concentration range from 0.5 mM to 16 mM). Kinetic parameters *k*_cat_ and *K*_m_ were obtained with the help of software Origin 8.5 by plotting enzymatic activity versus substrate concentrations and fitting them using the Michaelis–Menten equation. The parameter *k*_cat_ was then calculated according to the following equation: *k*_cat_ = *V*_*max*_/(E), in which (E) means the molar concentration of the enzymes as shown in Additional file 7: Figure S4 [33].

### Construction of recombinant *Pseudomonas putida* KT2440

One of the improved variants *styAB*_D305X_ (X = V, A, G) or the wild type were cloned, together with *fdh*, into the expression plasmid pJB861 and the recombinant plasmid pJB861/*styAB*_D305X_-*fdh* or pJB861/*styAB*-*fdh* was transformed into competent *Pseudomonas putida* KT2440 cells by electroporation in order to find the optimal system for biotransformation of the olefins and aromatic compounds [54]. All positive clones were sequenced by Sangon Biotech. The single colonies were grown over 24 h at 30 °C in EM medium containing kanamycin (50 μg/mL). One milliliter of the culture was then inoculated into 100 mL of EM medium, then cultured at 30 °C for 24 h to induce the protein expression. The cells were harvested by centrifugation at 8000×*g* for 8 min at 4 °C.

### Screening of organic solvents to construct a biphasic system

Effects of water-miscible and water-immiscible organic solvents on enzyme activity of SMO were determined to select suitable organic solvents for constructing a biphasic system (Additional file 2: Figure S2). The whole cell biotransformation was performed by using *Pseudomonas putida* KT2440/pJB861-*styAB*_D305G_-*fdh* in presence of styrene. The reaction was carried out in 50 mL flasks with 10 mL of KP buffer (200 mM, pH 8.0) containing 26 mM of the substrate styrene and biomass with a cell dry weight of 1.0 g. The cultures were incubated at 30 °C for 8 h. All the study conditions were the same except the presence of 10, 15, 20 or 30% (v/v) of ethanol, isopropanol, cyclodextrin or DMSO [55]. The reaction solution without adding any organic solvent was used as a control. Furthermore, the tolerance of SMO in different water-immiscible organic solvents was additionally tested using hexadecane, bis-(2-ethylhexyl) phthalate (BEHP), *n*-hexane, toluene, dichloromethane, ethyl acetate, trichloromethane and cyclohexane. The reaction mixture containing KP buffer and one of the water-immiscible organic solvent (1:1, v/v) was incubated at 30 °C for 8 h with shaking at 220 rpm. After incubation, the reaction mixture was terminated by centrifugation. The organic phase was analyzed by HPLC. For the aqueous phase, multiple extractions were performed to ensure complete separation of the product in the organic phase. KP buffer with enzyme, but without any pretreatment of the organic solvent, was used as control. Among all the organic solvents, there are 8 solvents which were used to construct biphasic systems for a biotransformation process at high substrate concentration. Reaction samples (100 μL) was withdrawn periodically and analyzed by reverse phase HPLC on a Luna C_18_ column at a flow rate of 0.8 mL/min under a methanol–water mixture at a ratio of 75:25.

## Additional files

**Additional file 1: Figure S3.** Effects of temperature and pH on SMO activity.

**Additional file 2: Figure S2.** Effect of water-miscible and water-immiscible organic solvents on SMO activity.

**Additional file 3: Figure S6.** Time course of biotransformation by the recombinant *Pseudomonas putida* KT2440/pJB861-*styAB*_D305G_-*fdh* cells from styrene to styrene oxide.

**Additional file 4: Table S1.** Strains and plasmids used in this work.

**Additional file 5: Figure S1.** A section of a multiple-sequence alignment of *styA* with oxygenases from diverse proteins.

**Additional file 6: Figure S5.** Biotransformation of 1a-4a to (*S*)-1d-4d by the whole cell of recombinant *Pseudomonas putida* KT2440.

**Additional file 7: Figure S4.** The Michaelis-Menten Plots.

## Abbreviations

epPCR: error-prone PCR
CDW: cells dry weight
KP buffer: potassium phosphate buffer
