# Supplementary material for: Asp305Gly mutation improved the activity and stability of the styrene monooxygenase for efficient epoxide production in Pseudomonas putida KT2440
Source: Microb Cell Fact. 2019 Jan 24;18:12. doi: 10.1186/s12934-019-1065-5 (PMC6345017; doi:10.1186/s12934-019-1065-5)
Supplement: Supplementary file 1 — Additional file 1: Figure S3. Effects of temperature and pH on SMO activity. [file 12934_2019_1065_MOESM1_ESM.doc]

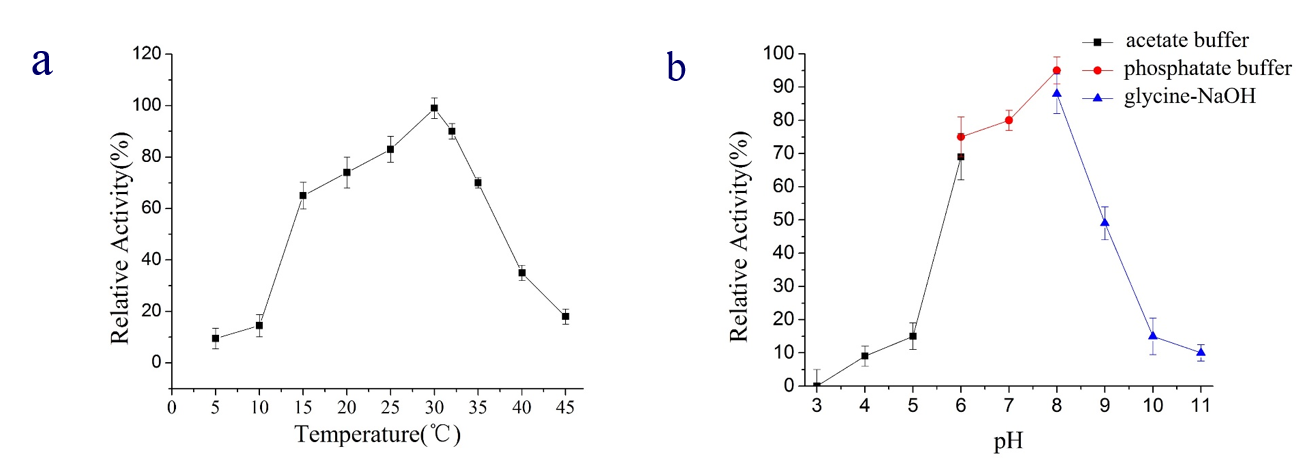
**Fig. S3 Effects of temperature and pH on SMO activity.** The reaction was in 2 mL volumes: 0.8 U/mL purified SMOA, 1.6 U/mL of purified SMOB, 1.7 U/mL formate dehydrogenase, 0.2M sodium formate, 0.3 mM NADH, 1mM NAD+, 0.05 mM FAD, and 200 mM styrene (from a 200-fold stock in ethanol). (a) The optimal temperature for the SMO. (b)The optimal pH for the SMO. The initial activity before incubation was set to 100%, corresponding to an initial activity of 5.6 ± 0.21 U/mg. All assays were performed in triplicate and the standard deviations of the biological replicates are represented by error bars.
