# Supplementary material for: Asp305Gly mutation improved the activity and stability of the styrene monooxygenase for efficient epoxide production in Pseudomonas putida KT2440
Source: Microb Cell Fact. 2019 Jan 24;18:12. doi: 10.1186/s12934-019-1065-5 (PMC6345017; doi:10.1186/s12934-019-1065-5)
Supplement: Supplementary file 2 — Additional file 2: Figure S2. Effect of water-miscible and water-immiscible organic solvents on SMO activity. [file 12934_2019_1065_MOESM2_ESM.doc]

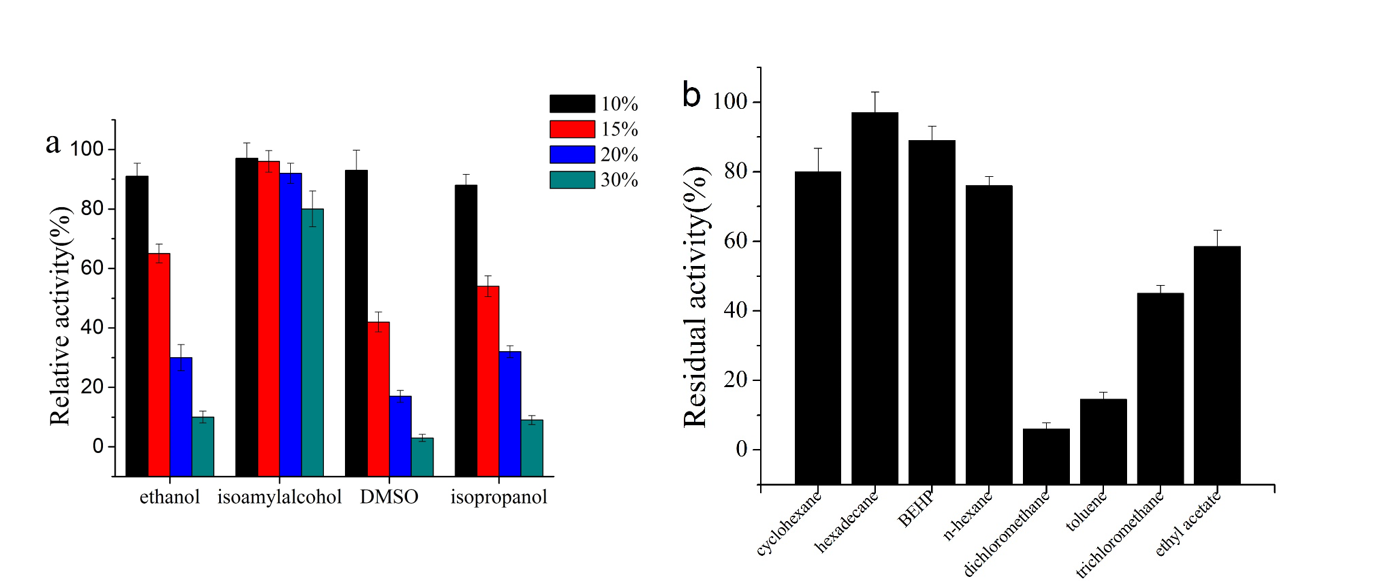


**Fig. S2 Effect of water-miscible and water-immiscible organic solvents on SMO activity.** The whole cell biotransformation was conducted by *Pseudomonas putida* KT2440/pJB861- *styAB*_D305G_-*fdh* toward styrene. Reaction mixture was incubated at 30 °C and 220 rpm for 8 h. 100 % corresponds to an initial activity of 72 ± 10 U/g CDW. All assays were performed in triplicate.
