# Supplementary material for: Asp305Gly mutation improved the activity and stability of the styrene monooxygenase for efficient epoxide production in Pseudomonas putida KT2440
Source: Microb Cell Fact. 2019 Jan 24;18:12. doi: 10.1186/s12934-019-1065-5 (PMC6345017; doi:10.1186/s12934-019-1065-5)
Supplement: Supplementary file 3 — Additional file 3: Figure S6. Time course of biotransformation by the recombinant Pseudomonas putida KT2440/pJB861-styABD305G-fdh cells from styrene to styrene oxide. [file 12934_2019_1065_MOESM3_ESM.doc]

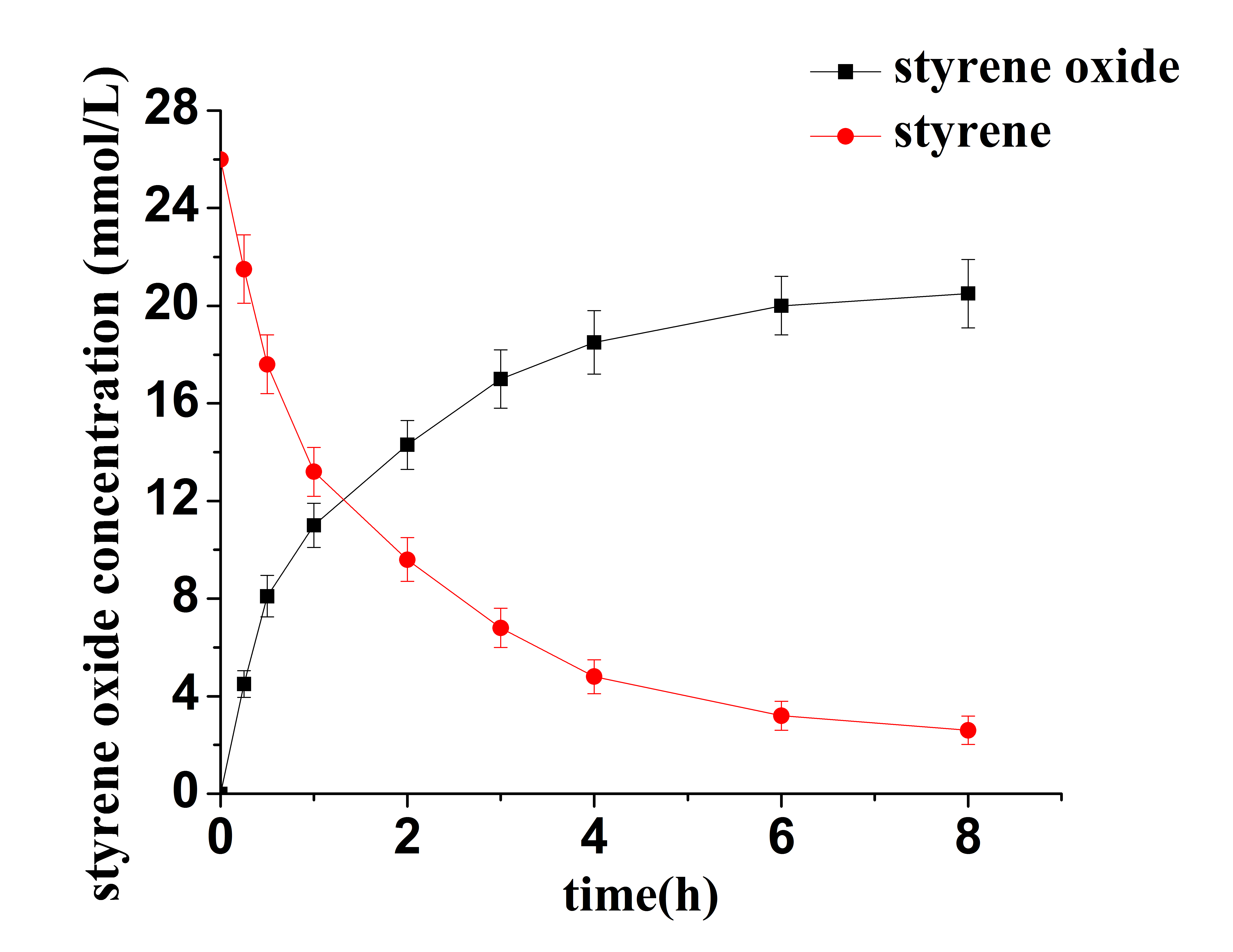


**Fig. S6. Time course of biotransformation by the recombinant *Pseudomonas putida* KT2440/pJB861-*styAB*_D305G_-*fdh* from styrene to styrene oxide.** The whole cell biotransformation was carried out in 50 mL flasks with 10 ml of 200 mM KP buffer pH=8.0 containing 26 mM of styrene and 1.0 g cell dry weight with addition of 50% (v/v) hexadecane at 30 °C and 220 rpm on a rotatory shaker for 8 h. The product was withdrawn periodically and analyzed by reverse phase HPLC on a Luna C_18_ column at a flow rate of 0.8 mL/min under a methanol–water mixture at a ratio of 75:25. All assays were performed in triplicate and the standard deviations of the biological replicates are represented by error bars.
