# Supplementary material for: Asp305Gly mutation improved the activity and stability of the styrene monooxygenase for efficient epoxide production in Pseudomonas putida KT2440
Source: Microb Cell Fact. 2019 Jan 24;18:12. doi: 10.1186/s12934-019-1065-5 (PMC6345017; doi:10.1186/s12934-019-1065-5)
Supplement: Supplementary file 4 — Additional file 4: Table S1. Strains and plasmids used in this work. [file 12934_2019_1065_MOESM4_ESM.doc]

**Table S1 Strains and plasmids used in this work.**

| **Strains /plasmids** | **Characteristics** | **Source** |
| --- | --- | --- |
| **Strains**  *E. coli* Bl21 (DE3)  *E. coli* JM109  *Pseudomonas putida* KT2440  **Plasmids**  pET28a-*styA*  pET28a-*styA*  pET28a-*fdh*  pET28a-*styA* _D305X_  pJB861-*styAB*  pETDuet-1-*styAB*  pJB861-*styab*_D305G_-*fdh*  pJB861-*styab*_D305V_-*fdh*  pJB861-*styab*_D305A_-*fdh* | Host for gene expressing  Host for gene cloning  Host for gene expressing  Kan^R^, expression SMOA in *E. coli*  Kan^R^, expression SMOB in *E. coli*  Kan^R^, expression FDH in *E. coli*  Kan^R^, expression SMOA in *E. coli*  Kan^R^, expression SMO in *Pseudomonas putida* KT2440  Amp^R^, expression SMO in *E. coli*  Kan^R^, expression SMO in *Pseudomonas putida* KT2440  Kan^R^, expression SMO in *Pseudomonas putida* KT2440  Kan^R^, expression SMO in *Pseudomonas putida* KT2440 | Laboratory  Laboratory  Laboratory  This study  This study  This study  This study  This study  This study  This study  This study  This study |
