# Supplementary material for: Asp305Gly mutation improved the activity and stability of the styrene monooxygenase for efficient epoxide production in Pseudomonas putida KT2440
Source: Microb Cell Fact. 2019 Jan 24;18:12. doi: 10.1186/s12934-019-1065-5 (PMC6345017; doi:10.1186/s12934-019-1065-5)
Supplement: Supplementary file 5 — Additional file 5: Figure S1. A section of a multiple-sequence alignment of styA with oxygenases from diverse proteins. [file 12934_2019_1065_MOESM5_ESM.doc]

**Additional Information**

**Journal:** Microbial Cell Factories

**Title:** Asp305Gly mutation improved the activity and stability of the styrene monooxygenase for efficient epoxide production in Pseudomonas putida KT2440.

**Authors:** Chunlin Tan^a^, Xian Zhang^a^, *, Zhijing Zhu^b^, Meijuan Xu^a^, Taowei Yang^a^, Tolbert Osire^a^, Shangtian Yang^c^ , Zhiming Rao^a^, *

^a^ The Key Laboratory of Industrial Biotechnology, Ministry of Education, School of Biotechnology, Jiangnan University, Wuxi 214122, China

^b^ The school of digital media, Jiangnan University, Wuxi 214122, China

^c^ Department of Chemical and Biomolecular Engineering, The Ohio State University, Columbus, OH 43210, USA

*Corresponding author at: The Key Laboratory of Industrial Biotechnology of Ministry of Education, School of Biotechnology, Jiangnan University, 1800 Lihu Road, Wuxi, Jiangsu 214122, China.

Tel: +86-0510-85910886

E-mail: raozhm@jiangnan.edu.cn

**Additional Information:**


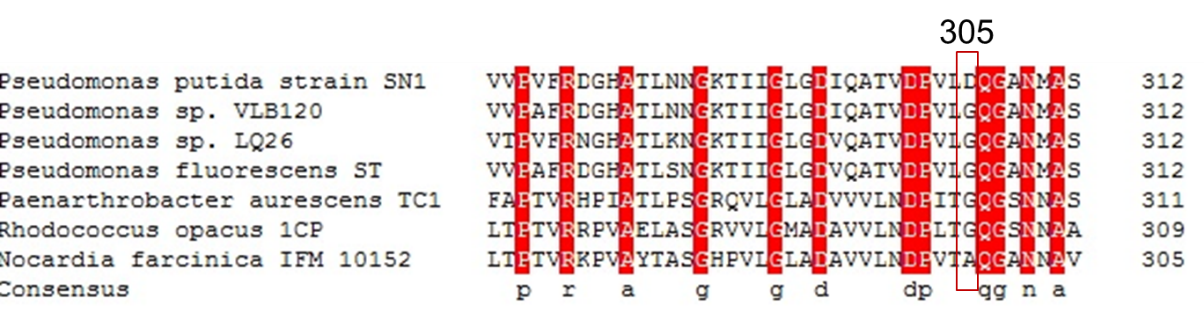


**Fig. S1 A section of a multiple-sequence alignment of *styA* with oxygenases from diverse proteins. It was performed by the software DNAMAN**. Amino acid residue at position 305 is bordered to show the natural existence of D305 in several SMOs which are from different sources.
