# Supplementary material for: Asp305Gly mutation improved the activity and stability of the styrene monooxygenase for efficient epoxide production in Pseudomonas putida KT2440
Source: Microb Cell Fact. 2019 Jan 24;18:12. doi: 10.1186/s12934-019-1065-5 (PMC6345017; doi:10.1186/s12934-019-1065-5)
Supplement: Supplementary file 6 — Additional file 6: Figure S5. Biotransformation of 1a-4a to (S)-1d-4d by the whole cell of recombinant Pseudomonas putida KT2440. [file 12934_2019_1065_MOESM6_ESM.doc]

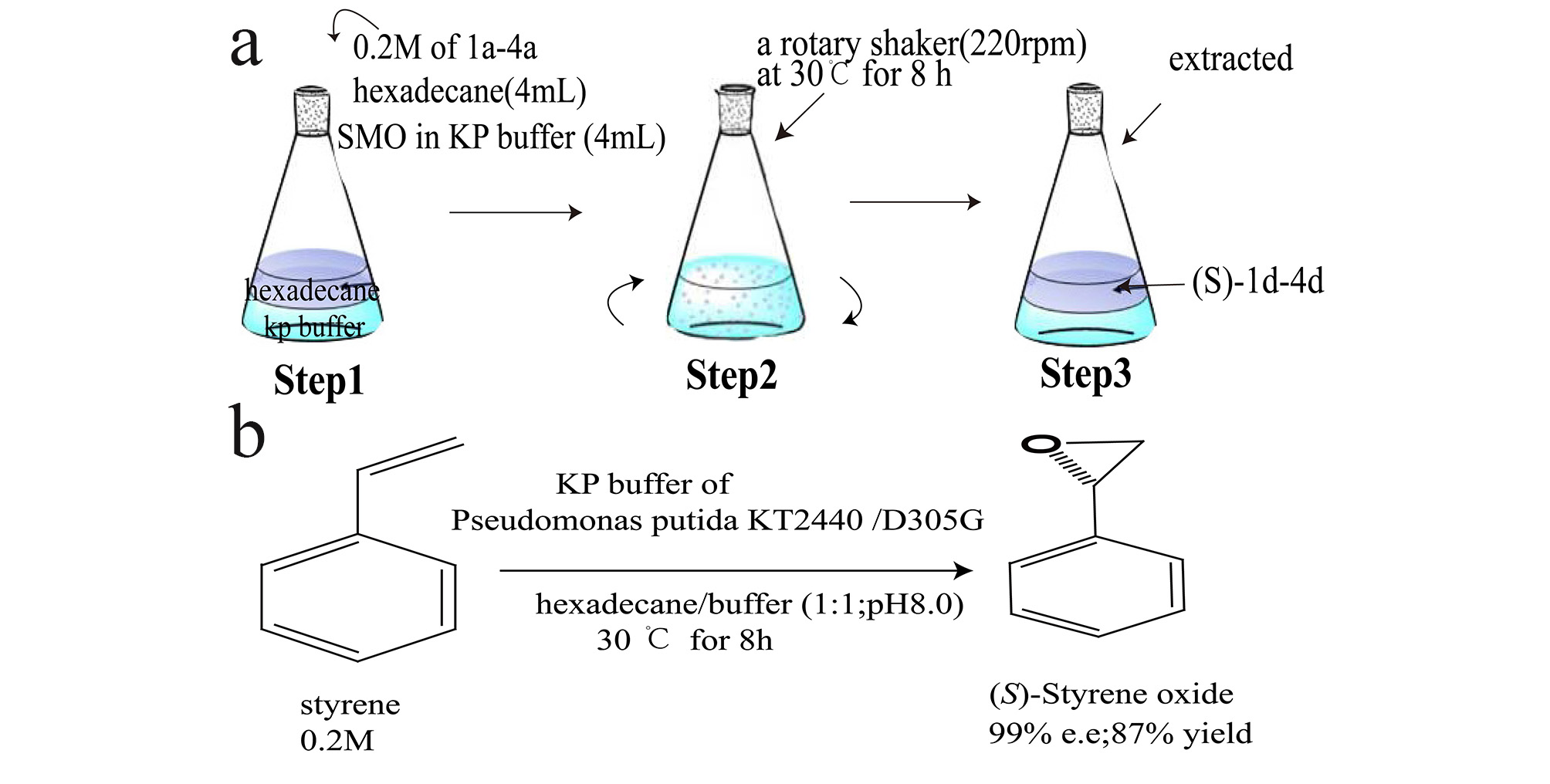


**Fig. S5** **Biotransformation of 1a-4a to (*S*)-1d-4d by the whole cell of recombinant *Pseudomonas putida* KT2440.** The reaction mixture containing one of alkene substrate 1a-4a (0.2 M) and the freshly prepared cells in a hexadecane/buffer biphasic system to a total volume 8 mL was incubated at 30 °C in a rotary shaker (220 rpm). The mixture without pretreatment with any organic solvent was used as a control.
