# Supplementary material for: Asp305Gly mutation improved the activity and stability of the styrene monooxygenase for efficient epoxide production in Pseudomonas putida KT2440
Source: Microb Cell Fact. 2019 Jan 24;18:12. doi: 10.1186/s12934-019-1065-5 (PMC6345017; doi:10.1186/s12934-019-1065-5)
Supplement: Supplementary file 7 — Additional file 7: Figure S4. The Michaelis-Menten Plots. [file 12934_2019_1065_MOESM7_ESM.doc]

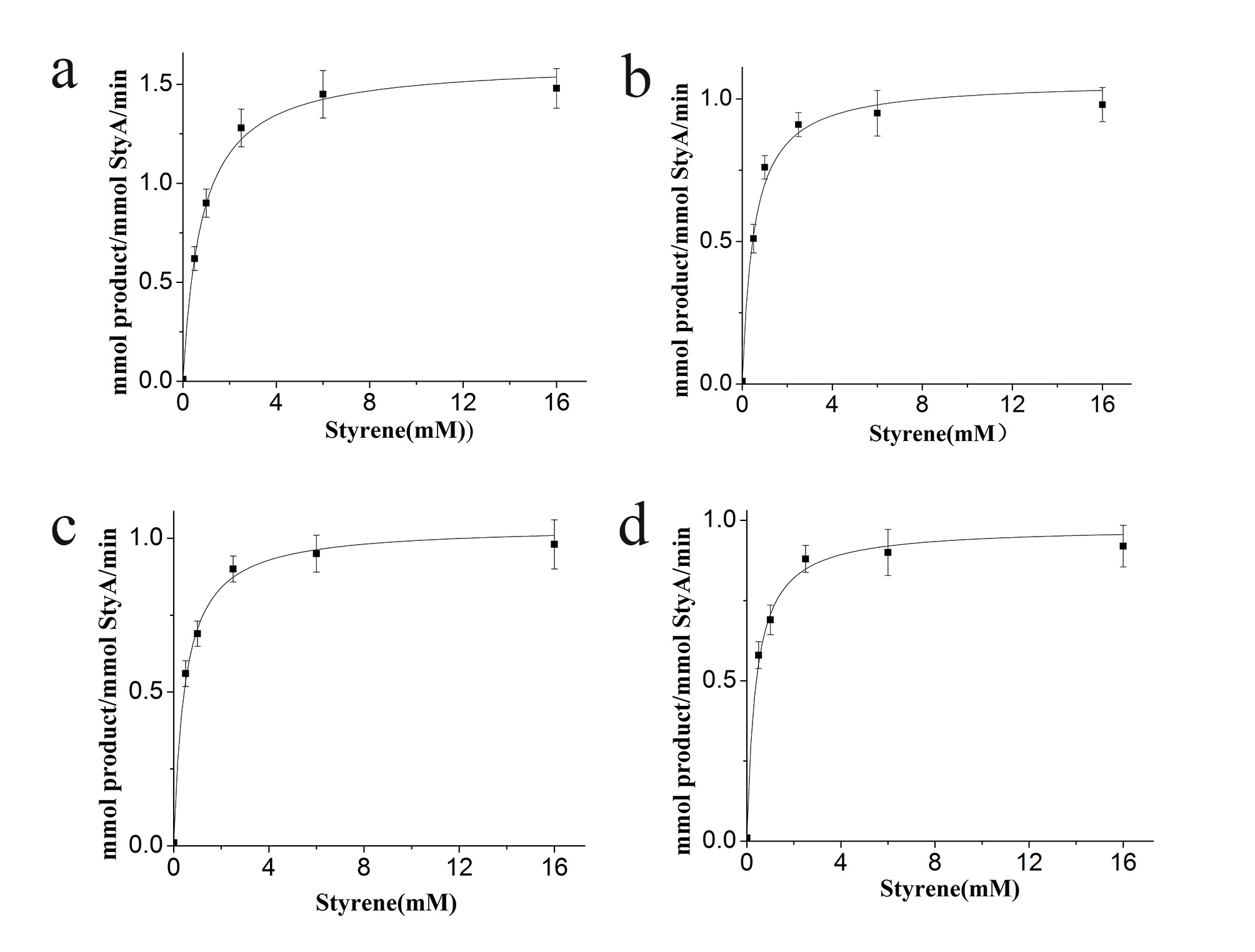


**Fig. S4 The Michaelis-Menten Plots:** (a) *K*_m_ of the wild type（b）*K*_m_ of the D305V (c) *K*_m_ of the D305A (d) *K*_m_ of the D305G. All assays were performed in triplicate and the standard deviations of the biological replicates are represented by error bars.
